# Relationship Between Plasma Adiponectin Level With Inflammatory and Metabolic Markers in Patients With Chronic Kidney Disease

**DOI:** 10.5812/numonthly.11743

**Published:** 2013-12-15

**Authors:** Omid Sedighi, Saeid Abediankenari

**Affiliations:** 1 Department of Nephrology, Imam Khomeini Hospital, Mazandaran University of Medical Sciences, Sari, IR Iran; 2 Diabetes Research Center, Mazandaran University of Medical Sciences, Sari, IR Iran

**Keywords:** Adiponectin, Kidney Failure, Chronic, Body Mass Index

## Abstract

**Background:**

Adiponectin (ADPN) is an important anti-inflammatory marker with anti-atherogenic effects. However, its role in patients with chronic kidney disease (CKD) should be determined.

**Objectives:**

The aim of this study was to determine the relationship between plasma adiponectin level with some inflammatory and metabolic markers in CKD patients.

**Patients and Methods:**

In this case-control study, we measured plasma ADPN level in 42 CKD patients and 46 healthy persons with the same age and sex as control group. Then, we investigated the association between plasma ADPN level with some inflammatory and metabolic determinants in CKD patients.

**Results:**

Plasma ADPN level was significantly higher in CKD patients than control group (P = 0.04). It was directly correlated with HDL-cholesterol (r = 0.599, P < 0.001) and serum creatinine levels (r = 0.675, P < 0.001) and inversely correlated with glomerular filtration rate (GFR) (r = -0.570, P < 0.001), body mass index (BMI) (r = -0.318, P = 0.04), C-reactive protein (CRP) (r = -0.548, P < 0.001) and fasting blood sugar (FBS) (r = -0.640, P < 0.001) in CKD patients.

**Conclusions:**

These findings suggested that plasma ADPN level is inversely associated with GFR and directly correlate with HDL-cholesterol and inversely with some, but not all metabolic factors of CKD patients who were not undergone dialysis.

## 1. Background

Adiponectin (ADPN) is an important protein that secrete by adipocytes. Plasma level of ADPN is suggested as a predictive marker of metabolic syndrome in patients with chronic kidney disease (CKD) who did not undergo dialysis (1). In some animal studies, ADPN has both anti-inflammatory and anti-oxidative properties. In mice, ADPN deficiency causes foot processes effacement and albuminuria which are reverted after ADPN therapy (2). In human, plasma ADPN level is usually lower in patients with normal renal function and higher in patients with cardiovascular risk factors, such as type 2 diabetes, obesity, and ischemic heart disease (3). However, despite the higher risk of metabolic syndrome in CKD patients (4), plasma level of ADPN is higher in pre-dialysis patients (5) and also patients on hemodialysis (HD) and peritoneal dialysis (PD) (6).

Higher plasma ADPN level in CKD patients depends on low glomerular filtration rate (GFR) (7) because, it significantly decreases after successful kidney transplantation (8). In CKD patients, the role of adiponectin in metabolic syndrome is unclear. In some studies, plasma ADPN level is directly associated with cardiovascular diseases (CVD) and mortality in predialysis CKD patients (9). However, in other studies low plasma ADPN level predicts high cardiovascular events in HD patients (6).

## 2. Objectives

The aim of our study was to determine the relationship between plasma ADPN level and some inflammatory, and metabolic factors in CKD predialysis patients.

## 3. Patients and Methods

### 3.1. Patients

In this case-control study, 42 CKD patients with a GFR level of 15-90 mL/min, referred to the nephrology clinic in Sari in Iran, were selected. GFR estimated by the modified diet in renal disease (MDRD) equation. This group of patients compared with 46 persons in control group with the same age and gender. Exclusions criteria were the presence of active infection, acute myocardial infarction in last 6 weeks, uncontrolled diabetes mellitus, consumption of antioxidant drugs, and presence of active inflammatory diseases. The study was in agreement with the ethics committee guidelines of our institution and all of the participating persons signed an informed consent form.

### 3.2. Laboratory Studies

Fasting blood specimens were collected from both patients and control groups and then the metabolic and inflammatory markers were measured. Blood urea nitrogen (BUN), creatinine, total cholesterol, high density lipoprotein (HDL) cholesterol, low density lipoprotein (LDL) cholesterol, triglyceride, fasting blood sugar (FBS), calcium, phosphorous, albumin (Alb) (Pars Azmun, Iran), erythrocyte sedimentation rate (ESR), C-reactive protein (CPR), and hemoglobin (Hb) levels were measured by standard kits and using an auto analyzer (prestige 24i, Japan). Body mass index (BMI) was calculated by dividing body weight (kg) to the height (m^2^). Plasma APDN level (all the isoforms) was measured using an ELISA kit (bender med system, Germany, CV = 2.9) in accordance to manufacture procedure.

### 3.3. Statistical Analysis

Data were expressed as the mean standard deviation and analyzed using SPSS software (version 17.0, SPSS Inc). Comparison between the patientsand the control group was performed by t-test (quantitative variables) and Chi-squared test (qualitative variables). P < 0.05 considered statistically significant.

## 4. Results

The baseline clinical characteristics of CKD patients (group I, n = 42) and healthy control (group II, n = 46) are shown in Table 1. Mean age of CKD patients and control groups were 65.19 ± 12.69 and 61.04 ± 8.23 years respectively. There were no significant differences between two groups based on the age, sex, and BMI. Mean plasma ADPN levels in group I was 17.84 ± 14.18 mg/L and in group II was 13.26 ± 6.63 mg/L. Plasma ADPN level was significantly higher in CKD patients than control group (P = 0.042). 

**Table 1.** Clinical Characteristics of CKD^a^ Patients (Group I, n = 42) and Healthy Control (Group II, n = 46)

Parameter	Group I (n = 42)	Group II (n = 46)	P Value
**Age, mean ± SD, y**	65.19 ± 12.69	61.41 ± 8.23	0.106
**Gender, M^a^/F^a^**	22/20	26/20	0.831
**BMI^a^, mean ± SD, kg/m^2^**	23.85 ± 2.87	23.98 ± 3.24	0.838
**GFR^a^, mean ± SD, mL/min**	42.57 ± 37.72	98.65 ± 18.45	< 0.001
**Serum Cr, mean ± SD, mg/dL**	2.24 ± 0.81	0.98 ± 0.15	< 0.001
**ADPN^a^, mean ± SD, mg/L**	17.84 ± 14.18	13.26 ± 6.63	0.042
**Cholesterol, mean ± SD, mg/dL**	202.54 ± 40.91	213.43 ± 45.47	0.243
**Triglyceride, mean ± SD, mg/dL**	198.54 ± 103.27	181.47 ± 94.10	0.422
**LDL^a^, mean ± SD, mg/dL**	112.74 ± 33.84	121.88 ± 24.28	0.149
**HDL^a^, mean ± SD, mg/dL**	40.30 ± 9.37	45.91 ± 11.94	0.055
**Alb^a^, mean ± SD, g/dL**	4.19 ± 0.71	4.65 ± 0.38	0.02
**CRP^a^, mean ± SD, mg/dL**	5.41 ± 3.67	3.39 ± 4.23	0.02
**Hb^a^, mean ± SD, g/dL**	11.76 ± 1.83	13.59 ± 1.46	0.001
**ESR^a^, mean ± SD, mm/h**	59.54 ± 14.62	22.65 ± 13.3	0.001
**FBS^a^, mean ± SD, mg/dL**	107.54 ± 35.57	95.63 ± 15.56	0.087
**Ca, mean ± SD, mg/dL**	9.01 ± 1.05	9.55 ± 0.42	0.003
**P, mean ± SD, mg/dL**	4.25 ± 1.11	3.65 ± 0.40	0.004

^a^ Abbreviations: ADPN, adiponectin; BMI, body mass index; CKD, chronic kidney disease; CRP, c-reactive protein; ESR, erythrocyte sedimentation rate; FBS, fasting blood sugar; F, female; GFR, glomerular filtration rate; Hb, hemoglobin; HDL, high density lipoprotein; LDL, low density lipoprotein; M, male.

Table 2 shows the correlation between plasma ADPN level and other clinical and biochemical profiles in CKD patients group. 

**Table 2.** Correlation of Plasma ADPN^a^ Level With Clinical and Biochemical Variables in CKD^a^ Patients

Parameter	Correlation	P Value
**Age, y**	-0.152	0.337
**BMI^a^, kg/m^2^**	-0.318	0.04
**GFR^a^, mL/min**	-0.570	< 0.001
**Serum Cr, mg/dL**	0.675	< 0.001
**Total cholesterol, mg/dL**	-0.271	0.083
**Triglyceride, mg/dL**	-0.182	0.248
**LDL^a^, mg/dL**	-0.189	0.230
**HDL^a^, mg/dL**	0.599	< 0.001
**CRP^a^, mg/dL**	-0.548	< 0.001
**ESR^a^, mm/h**	0.57	0.722
**Ca, mg/dL**	0.148	0.352
**P, mg/dL**	0.09	0.569
**Alb^a^, g/dL**	0.101	0.525
**Hb^a^, g/dL**	-0.05	0.751
**FBS^a^, mg/dL**	-0.640	< 0.001

^a^ Abbreviations: ADPN, adiponectin; BMI, body mass index; CKD, chronic kidney disease; CRP, c-reactive protein; ESR, erythrocyte sedimentation rate; FBS, fasting blood sugar; GFR, glomerular filtration rate; Hb, hemoglobin; HDL, high density lipoprotein; LDL, low density lipoprotein.

There was no significant correlation between plasma ADPN level and age (r = -0.152, P = 0.337), serum total cholesterol (r = -0.271, P = 0.083), triglyceride (r = -0.182, P = 0.248), LDL-cholesterol (r = -0.189, P = 0.230), albumin (r = 0.101, P = 0.525), and hemoglobin (r = -0.05, P = 0.751).

Plasma ADPN level was significantly and directly correlated with serum creatinine (r = 0.675, P < 0.001), and HDL-cholesterol levels (r = 0.599, P < 0.001), while it was significantly and inversely correlated with GFR (r = -0.570, P < 0.001), BMI (r = -0.378, P = 0.04), CRP (r = -0.548, P < 0.001), and FBS (r = -0.640, P < 0.001).

## 5. Discussion

We investigated the association between plasma ADPN level with some inflammatory and metabolic determinants in CKD patients. Several studies have shown that plasma ADPN level is inversely correlated with some metabolic syndrome factors such as BMI and type 2 diabetic mellitus in the general population (10). Other studies showed that plasma ADPN level is higher in CKD patients compared with healthy persons (11). The major mechanisms, can decrease the renal ADPN clearance (12), dysregulation in the ligand/receptor activity (13), or a response to metabolic disorders in renal dysfunction (14). Moreover, higher plasma ADPN level may be the predictor of disease progression in CKD patients (15).

Our study found that plasma ADPN level inversely correlates with GFR, some metabolic and inflammatory factors such as BMI, CRP, FBS, and HDL-cholesterol levels in CKD patients not in dialysis patients, but it was not associated with other important metabolic syndrome determinants such as total cholesterol, triglyceride, LDL-cholesterol, and other inflammatory markers such as ESR, albumin, and hemoglobin levels in these patients.

Adiponectin, an adipokine secreted by adipocyte, has many systemic effects and important roles in vascular protection. Hypoadiponectinemia is a risk factor for ischemic heart disease, hypertension and endothelial dysfunction in some studies (16). However, other studies had not found any association between low plasma ADPN level and cardiovascular mortality in the general population (17).

The role of ADPN in CKD patients is unclear. High plasma ADPN level was significantly correlated with a low risk of cardiovascular events in some studies (1, 11). Moreover, plasma ADPN level was inversely correlated with other metabolic syndrome determinants such as BMI, FBS, and Triglyceride. It also directly correlated with HDL in CKD patients did not undergo dialysis (5).

Anti-inflammatory effects of ADPN in CKD patients may be due to inhibition of oxidative stress or induction of macrophage tolerance stimulated by new inflammatory stimuli (18). Our study had some limitation. First, we measured the total plasma ADPN level while, APDN has at the least three different oligomeric complexes with multiple functions (19). However, analysis of total ADPN or a specific oligomeric complex in plasma has provided similar results (20). Second, we analyzed a small number of patients in a single center, so other multicenter studies with large number of patients are required to evaluate the role of ADPN in CKD patients, precisely.

In summary, the results of this study showed that the plasma ADPN was inversely correlated with some determinants of the metabolic syndrome. In addition, plasma total ADPN level was inversely associated with GFR in these patients. Thus, it is concluded that ADPN can be known as an important marker for CKD patients.
